# Feasibility assessment of catheter-free water vapor thermal therapy for treatment of benign prostatic hyperplasia

**DOI:** 10.1007/s00345-024-05002-4

**Published:** 2024-06-21

**Authors:** Vi Nguyen, Joshua Winograd, Alia J. Codelia-Anjum, Kevin C. Zorn, Dean Elterman, Naeem Bhojani, Seth K. Bechis, Bilal Chughtai

**Affiliations:** 1 Department of Urology, University of California, 9333 Genesee Avenue, Suite 320, La Jolla, CA 92121 USA; 2 Department of Urology, Weill Cornell Medical College/New York Presbyterian, New York, NY USA; 3 Department of Urology, University of Montreal Hospital Center, Montreal, QC Canada; 4 Division of Urology, Department of Surgery, University Health Network, University of Toronto, Toronto, ON Canada; 5 Department of Urology, New Hyde Park, Northwell Health, NY USA

**Keywords:** Water vapor thermal therapy, Rezum, Benign prostatic hyperplasia

## Abstract

**Purpose:**

To investigate safety and feasibility of performing water vapor thermal therapy (WVTT; Rezum, Boston Scientific, Marlborough, MA, USA) without postoperative catheterization among men with benign prostatic hyperplasia.

**Methods:**

This is a prospective, single arm, unblinded pilot study of 20 consecutive male patients ages 40–80 who underwent WVTT at a single academic institution. All patients underwent 1 injection per lobe at the point of maximal obstruction based on visualization. Primary outcome was evaluation of voiding parameters, symptom scores, and need for catheterization at 3 day, 1, 3, and 6 month follow up compared to baseline visit 30 days prior to surgery.

**Results:**

Mean age was 65 years (range 55–75). Mean prostate volume and PVR were 43 cc (range 30–68) and 89 cc, with 30% (*n* = 6) having median lobes. Patients received 2–3 treatments based on presence of bilobar versus trilobar hyperplasia. One patient (55 cc prostate, no median lobe) required catheterization for acute urinary retention on postoperative day 2. No patients required antibiotics for urinary tract infection or inpatient readmission within 30 days. Qmax significantly increased from 6 mL/s to 8, 13, 12, and 14 at 3 days, 1, 3, and 6 months (*p* < 0.05). IPSS decreased from 17 preoperatively to 10, 6, 7, and 8 (*p* < 0.05). No significant differences were noted in PVR, IIEF, MSHQ-EjD, or SF-12.

**Conclusions:**

In well-selected men, catheter-free WVTT is feasible and improved voiding parameters and symptom scores. No changes in sexual function, infectious complications, or readmission were noted. Only 1 patient (5%) required postoperative catheterization within 30 days.

**Supplementary Information:**

The online version contains supplementary material available at 10.1007/s00345-024-05002-4.

## Introduction

Benign prostatic hyperplasia (BPH) is a common cause of bladder outlet obstruction, occurring secondary to proliferation of glandular epithelial tissue, smooth muscle, and connective tissue in the transition zone of the prostate. Clinically, BPH can manifest as lower urinary tract symptoms (LUTS) including storage symptoms such as urgency, frequency, and nocturia, as well as voiding symptoms such as straining, hesitancy, incomplete emptying and weak or intermittent stream [1, 2]. BPH is intimately associated with the aging male population, with a 60% prevalence in men over age 60 and 80% prevalence in men over age 80 [1, 2]. Several surgical treatments are available, particularly with the recent emergence of minimally invasive surgical therapies (MIST) [2].

Water vapor thermal therapy (WVTT; Rezum, Boston Scientific, Marlborough, MA, USA) is a MIST that utilizes the convective properties of water vapor to enable transurethral transfer of thermal energy to disrupt cell membranes and induce cell death [3–5]. One desirable aspect of WVTT is the preservation of erectile and ejaculatory function [6].However, one limitation of this procedure is the need for discharge home with foley catheter for an average duration of 3 to 5 days[7].

The objective of the present study is to investigate the feasibility of performing WVTT without the need for postoperative catheterization.

## Methods

### Study design and objective

This is a prospective, single institution, single arm, unblinded pilot study of 20 consecutive male patients ages 40–80 who underwent WVTT at an academic center. Institutional Review Board approval was obtained (IRB #20–02021474). The objective of this pilot study is to determine the feasibility and safety of performing WVTT without postoperative catheterization among men with BPH.

### Participant selection

Inclusion criteria for participant selection included ability to provide informed consent and ability to complete self-administered questionnaires, diagnosis of bladder outlet obstruction (BOO) secondary to BPH, surgical candidacy for WVTT, maximum flow rate (Qmax) < 15 mL/s, prostate volume 30–80 cc by transrectal ultrasound (TRUS), normal serum creatinine, and ability to perform intermittent catheterization. All patients underwent urodynamics and had to have bladder contractility index (BCI) score ≥ 100 (calculated by detrusor pressure Pdet Qmax + 5Qmax) to exclude those with detrusor underactivity. Other exclusion criteria included life expectancy less than 2 years, enrollment in concurrent separate drug or device study, active infection, diagnosis of chronic prostatitis or chronic pelvic pain, diagnosis of urethral stricture or bladder neck contracture within the last 180 days, diagnosis of 2 or more urethral strictures or bladder neck contractures within the last 5 years, lichen sclerosis, neurogenic bladder, polyneuropathy, history of lower urinary tract surgery, stress urinary incontinence, catheterization or post-void residual (PVR) > 400 ml in the 14 days prior to WVTT, and current diagnosis of bladder stones.

### Participant follow up

Patient visits and associated assessments are outlined in Table 1. Preoperatively, each patient underwent a baseline visit 30 days prior to WVTT. Intraoperatively, all patients underwent 1 injection per lobe at the point of maximal obstruction based on visualization. All procedures were carried out by one surgeon at a single academic center. This surgeon performs 5–6 cases of WVTT per month. Procedures were done in clinic with local anesthetic using a prostate block. Patients were positioned in lithotomy. Postoperative visit was scheduled 3 days after the procedure, with subsequent follow up at 1, 3, and 6 months. Unscheduled visits for any adverse events were also documented. Patients were designated as lost to follow up if they failed to return for visit after three attempts to contact. Subjects were able to voluntarily withdraw from study at any point. Other indications for participant discontinuation include disease progression requiring clinical indication for catheter, adverse event that is life threatening or requires hospitalization, or emergence of confounding illness.

**Table 1:** Schedule of Assessments

Evaluation	Baseline	Treatment	Postoperative	1 Month	3 Months	6 Months	Unscheduled
Allowable Window	30 days	N/A	3 days	+ 5 days	+ 14 days	+ 14 days	N/A
Informed Consent	X						
Inclusion / Exclusion Criteria	X						
Medical History	X						
Physical Examination / Vital Signs	X	X (vitals only)			X	X	
UDS / Urocuff	X				X	X	
CBC / CMP	X	X					X
PSA	X						
Urinalysis and Urine Culture	X	X					X
Uroflow / PVR	X		X	X	X	X	X
Cystoscopy		X					
TRUS	X					X	
Rezum		X					
IPSS	X			X	X	X	X
IIEF	X			X	X	X	X
MSHQ	X			X	X	X	X
OAB-SF	X			X	X	X	X
SF-12	X			X	X	X	X
Concomitant Medications	X	X		X	X	X	X
Adverse Events		X		X	X	X	X

### Outcomes

The primary outcome of this study was evaluation of need for catheterization for postoperative acute urinary retention, as well as voiding parameters (voided volume, Qmax, and PVR), International Prostate Symptom Score (IPSS). Secondary outcomes included changes in the International Index of Erectile Function (IIEF), Male Sexual Health Questionnaire Ejaculatory Dysfunction (MSHQ-EjD), Overactive Bladder Short Form (OAB-SF), and Short-Form 12 (SF-12) at 3 days, 1, 3, and 6 months postoperatively.

### Statistical analysis

Voided volume, Qmax, and PVR were compared to baseline using paired t-tests. All symptom scores (IPSS, IIEF, MSHQ-EjD, OAB-SF, and SF-12) were compared using paired Wilcoxon signed-rank tests. All other timepoints were compared to the preoperative baseline.

## Results

Mean age of the cohort was 65 ± 6 years (range 55–75). Mean prostate volume was 43 cc (range 30–68). 30% of patients (*n* = 6) had a median lobe on cystoscopy. Every patient underwent WVTT in the outpatient clinic setting under local anesthesia. 70% of patients underwent 2 injections, whereas the remaining 30% underwent a third injection due to presence of median lobe.

One patient went into acute urinary retention on postoperative day 2 requiring catheterization. A separate patient endorsed gross hematuria postoperatively which self-resolved without intervention or catheterization. No patients required antibiotic prescription for postoperative urinary tract infection. No patients were readmitted within 30 days after WVTT.

Table 2 summarizes changes in voiding parameters preoperatively as compared to postoperatively. The voided volume increased from a baseline of 89 mL to 129, 166, 197, and 189 mL at 3 days (*p* = 0.078), 1 month (*p* = 0.002), 3 months (*p* = 0.020), and 6 months (*p* = 0.031) postoperatively. Similarly, Qmax also increased from a baseline of 6 mL/s to 8, 13, 12, and 14 at 3 days (*p* = 0.163), 1 month (*p* < 0.001), 3 months (*p* = 0.046), and 6 months (*p* = 0.003) postoperatively. No significant differences were observed in PVR, which was overall low and fluctuated from a baseline of 89 mL to 77, 83, 91, and 57 at 3 days (*p* = 0.549), 1 month (*p* = 0.403), 3 months (*p* = 0.978), and 6 months (*p* = 0.164).

**Table 2:** Voiding parameters compared at baseline to 3 days, 1 month, 3 months, and 6 months postoperatively

Voided Volume (mL)	Median	Std Error	*t* score	*p*-value
Baseline	89	20.7		
3 days	129	53.1	−1.52	0.078
1 months	166	34.3	−3.53	**0.002**
3 months	197	53.6	−2.30	**0.020**
6 months	189	46.3	−2.06	**0.031**
Qmax (mL/s)				
Baseline	6	1.0		
3 days	8	1.5	−1.02	0.163
1 months	13	2.0	−4.01	** < 0.001**
3 months	12	1.7	−1.84	**0.046**
6 months	14	2.1	−3.39	**0.003**
PVR (mL)				
Baseline	89	26.3		
3 days	77	13.5	0.62	0.549
1 months	83	13.5	0.88	0.403
3 months	91	36.3	−0.03	0.978
6 months	57	22.1	1.63	0.164

The bold values represent significant *p*-values < 0.05

There were significant decreases in OAB-SF and IPSS symptom scores across all timepoints (Table 3). The OAB-SF decreased from a preoperative median of 45 to a median of 28, 29, 24, and 25 at 3 days (*p* = 0.008), 1 month (*p* = 0.012), 3 months (*p* = 0.023), and 6 months (*p* = 0.063) postoperatively. IPSS scores decreased from a median of 17 preoperatively to 10, 6, 7, and 8 at 3 days (*p* = 0.020), 1 month (*p* = 0.004), 3 months (*p* = 0.004), and 6 months (*p* = 0.031) postoperatively. Furthermore, significant changes in both the irritative and obstructive IPSS subscores were seen at nearly all timepoints, indicating improvement in both areas, as seen in Table S1.

**Table 3:** Symptom scores compared at baseline to 3 days, 1 month, 3 months, and 6 months postoperatively

OAB-SF	Median	Std Error	Wilcoxon Stat	*p*-value
Baseline	45	3.8		
3 days	28	4.9	1	**0.008**
1 months	29	6.3	0	**0.012**
3 months	24	3.9	2	**0.023**
6 months	25	3.7	0	0.063
IPSS				
Baseline	17	2.2		
3 days	10	2.0	3	**0.020**
1 months	6	1.9	0	**0.004**
3 months	7	2.2	0	**0.004**
6 months	8	2.6	0	**0.031**
IIEF				
Baseline	46	7.4		
3 days	34	7.4	10	0.263
1 months	47	8.6	14	0.359
3 months	41	7.1	11	0.203
6 months	48	10.3	1	0.063
MSHQ-EjD				
Baseline	11	0.8		
3 days	12	1.1	8	0.596
1 months	12	1.7	23	1.000
3 months	13	0.6	4	0.109
6 months	11	1.1	3	0.461
SF-12				
Baseline	34	0.6		
3 days	32	2.1	10	0.490
1 months	34	0.9	3	0.114
3 months	33	1.2	4	0.713
6 months	32	0.6	4	0.705

The bold values represent significant *p*-values < 0.05

No significant changes in IIEF, MSHQ-EjD, nor SF-12 were observed (Table 3).

## Discussion

WVTT is a MIST available for surgical management of BPH. However, one disadvantage of this procedure is the need for postoperative catheterization. Thus, the objective of this pilot study was to assess the feasibility and safety of performing this procedure without this standard pathway to identify the rate of postoperative urinary retention. We also evaluated voiding parameters and subjective symptom scores amongst our cohort.

This is the first study to assess a novel postoperative pathway of catheter-free WVTT, which results in improved objective voiding parameters (void volume and Qmax) as well as subjective symptom scores (IPSS and OAB-SF), without changes in sexual function. The postoperative catheterization rate within 30 days among our cohort was 5%. No patients required antibiotic treatment for urinary tract infection and no patients were readmitted within 30 days of their procedure.

Benefits garnered from lack of postoperative catheterization include increased patient comfort and decreased risk of urinary tract infection. The incidence of catheter-related bladder discomfort is estimated to range between 47% and 90%, manifesting as a burning sensation from the suprapubic to penile area associated with urge to void (Bai et al.) [8]. Furthermore, a retrospective cohort study analyzing Medicare patients from the National Surgical Infection Prevention Project demonstrated that postoperative catheterization longer than 2 days was associated with significantly increased likelihood of urinary tract infection and 30-day mortality (Wald et al.) [9].

According to the AUA guidelines, WVTT is recommended for prostate volume 30–80 cc [1, 2]. The mean prostate volume for our cohort was 43 cc, which falls within this recommendation. Although the guidelines do not recommend temporary implanted prostatic devices in patients with median lobes, median lobe is not an exclusion criterion for WVTT. Concordantly, 30% of our patients had median lobes. Of note, the patient who went into acute urinary retention on postoperative day 2 did not have a median lobe.

Our results are not generalizable to patients with baseline urinary retention, as the baseline PVR for our cohort was 89 mL, and we excluded patients with catheterization or PVR > 400 ml in the 14 days prior to WVTT. However, the standard WVTT pathway with postoperative catherization has been shown to be successful in liberating patients from catheter dependence. A retrospective review of WVTT among 49 patients with urinary retention with median PVR 900 mL (range 566–1146) demonstrated that 12.2% remained in retention at 6 months postoperatively (Bassily et al.). [10] Likewise, another retrospective study of WVTT among 136 men in urinary retention (mean catheter dependency duration 4.8 months) demonstrated a 94% and 91% rate of catheter-independence at 3 and 12 month follow up respectively (Eredics et al.) [11]. Another prospective study of 16 men with catheter dependence demonstrated a catheter-free rate of 93% at 1 month (Elterman et al.) [12]. However, the feasibility of upfront catheter-free WVTT among patients with baseline retention remains unknown.

In addition, all patients underwent urodynamics to establish bladder function in order to exclude patients with underlying detrusor underactivity. A prospective study of 32 patients with detrusor underactivity who underwent transurethral resection of prostate demonstrated significant improvement in IPSS and Qmax in all patients; however, 44% still required clean intermittent catheterization at 6 month follow up (Abdelhakim et al.) [13]. The efficacy of WVTT among patients with detrusor underactivity remains to be explored.

Lastly, all patients in our cohort received 2–3 injections (one in each lobe). Recent studies have demonstrated the safety and efficacy of the “less is more” approach, which minimizes the number of injections administered during WVTT (Babar et al. and Aladesuru et al.) [14, 15]. An area of future study would be to evaluate if the number of injections received among patients who undergo catheter-free WVTT is correlated to retention requiring postoperative catheterization. Long-term data is needed to assess the durability of this approach.

There were several limitations to our study, the first being the small sample size of 20 patients. The objective was to first assess feasibility of catheter-free WVTT, with the intention that if this approach was safe, this study would serve as the pilot for future studies. Furthermore, the unblinded design subjects the study to bias. Addition of a control arm would enable us to compare outcomes between patients who receive a catheter versus those who do not after WVTT. We recognize that our cohort focused on well-selected patients with small prostate volume, low baseline PVR, and lack of detrusor underactivity. Consequently, the selection criteria such as including urodynamics for each patient were more conservative and may not be generalizable to the typical BPH Urology practice. However, the data from this study is important as it forms the basis for further studies to examine larger cohorts of BPH patients. We are currently planning for a multi-institutional study with more generalizable patient selection criteria.

## Conclusions

Catheter-free WVTT is feasible in well-selected men with BPH and improved voiding parameters and symptom scores. No changes in sexual function, infectious complications, or readmission were noted. Only 1 patient (5%) required postoperative catheterization within the first 30 days. To the best of our knowledge, this is the first reporting of such postoperative management in a cohort of men with small prostate volume, low baseline PVR, and favorable preoperative detrusor function. Further study with larger sample size at the multi-institutional level is needed to validate our preliminary findings.

## Supplementary Information

Below is the link to the electronic supplementary material.

Supplementary file1 (DOCX 13 KB)

## Data Availability

Data available from authors upon request.
