# Supplementary material for: Feasibility assessment of catheter-free water vapor thermal therapy for treatment of benign prostatic hyperplasia
Source: World J Urol. 2024 Jun 21;42(1):383. doi: 10.1007/s00345-024-05002-4 (PMC11192826; doi:10.1007/s00345-024-05002-4)
Supplement: Supplementary file 1 — Supplementary file1 (DOCX 13 KB) [file 345_2024_5002_MOESM1_ESM.docx]

**Supplementary Table 1:** IPSS Obstructive and Irritative sub score analysis across all timepoints.

| **Obstructive** | **Median** | **Wilcoxon Stat.** | **p-value** |
| --- | --- | --- | --- |
| Baseline | 9 |  |  |
| 3 days | 6 | 6 | **0.014** |
| 1 months | 5 | 0 | **0.008** |
| 3 months | 3 | 0 | **0.012** |
| 6 months | 6 | 0 | **0.031** |
| **Irritative** |  |  |  |
| Baseline | 13 |  |  |
| 3 days | 5 | 3 | **0.005** |
| 1 months | 3 | 0 | **0.008** |
| 3 months | 4 | 3 | **0.020** |
| 6 months | 3 | 1 | 0.063 |
